# The Predictive Value of Tumor Necrosis Factor Receptor-Associated Factor-Interacting Protein With Forkhead-Associated Domain (TIFA) and Interleukin-1 Beta in Sepsis-Associated Acute Kidney Injury: Bioinformatics Analysis and Experimental Validation

**DOI:** 10.7759/cureus.84333

**Published:** 2025-05-18

**Authors:** Zuyi Zhao, Wen Guo, Bozhi Zhao, Long Ma

**Affiliations:** 1 Intensive Care Unit, The First Affiliated Hospital of Xinjiang Medical University, Urumqi, CHN

**Keywords:** geo, il-1β, sa-aki, sepsis, tifa

## Abstract

Background and objective

Sepsis is a systemic inflammatory response syndrome caused by severe infection. Sepsis-associated acute kidney injury (SA-AKI) is one of the most common complications of sepsis. Early prediction and subsequent treatment of SA-AKI can improve patient outcomes; hence, the accurate prediction of its occurrence is of paramount importance. This study aimed to investigate the predictive value of the potential biomarkers interleukin-1 beta (IL-1β) and tumor necrosis factor receptor‑associated factor (TRAF)‑interacting protein with forkhead‑associated domain (TIFA) related to the development of SA-AKI.

Methods

We identified relevant GSE datasets (225192) from the Gene Expression Omnibus (GEO) database and conducted secondary analyses, revealing increased expression of TIFA and IL-1β in renal tissues. Building on our preliminary findings, we performed a prospective observational study (March 2024 to December 2024) among patients with sepsis who were admitted to the Department of Critical Care Medicine at the First Affiliated Hospital of Xinjiang Medical University. Patients were stratified based on the development of AKI. Plasma samples were collected within 24 hours of ICU admission and analyzed using enzyme-linked immunosorbent assay (ELISA) to measure plasma levels of TIFA and IL-1β.

Results

The analysis revealed that the length of hospital stay, albumin/globulin ratio, and white blood cell count did not show any significant differences between groups. However, plasma levels of TIFA and IL-1β were significantly higher in patients with AKI compared to those without AKI. The area under the receiver operating characteristic (ROC) curve (AUC) was 0.912, indicating that TIFA and IL-1β possess high discriminatory power and calibration accuracy. These findings suggest that plasma levels of TIFA and IL-1β are closely associated with respect to the prediction of AKI in patients.

Conclusions

Bioinformatics analysis and experimental validation revealed that the expression levels of TIFA and IL-1β are significantly upregulated in patients with SA-AKI. These findings suggest that TIFA and IL-1β may serve as potential biomarkers for predicting SA-AKI.

## Introduction

Sepsis is typically characterized by a dysregulated host response to infection, manifesting in a multitude of signs and symptoms that are often nonspecific. This condition frequently progresses to life-threatening organ dysfunction. Globally, over 48 million individuals suffer from sepsis annually, with a mortality rate exceeding 20%, a proportion that is even higher in developing countries [1]. Sepsis is a leading cause of AKI (AKI) among critically ill patients, accounting for 45-70% of all AKI cases [2-4]. Sepsis-associated acute kidney injury (SA-AKI) exhibits a worse prognosis compared to sepsis or AKI alone [5-6]. Moreover, SA-AKI is associated with prolonged ICU and hospital stays, increased mortality, higher rates of long-term disability, and reduced quality of life in both adults and children [7]. An integrated approach involving early identification, targeted interventions, and close monitoring holds significant importance for alleviating the burden of SA-AKI and improving patient outcomes in the intensive care setting. Currently, numerous studies have focused on the prediction of AKI using sepsis-related biomarkers [8-14]. However, the sensitivity and specificity of these biomarkers are highly variable.

Vascular dysfunction, inflammatory storm, and autoregulatory failure constitute the pathophysiological foundation of sepsis. The initial innate immune response in sepsis is triggered by exposure to damage-associated molecular patterns (DAMPs), leading to the transcription and release of type I interferon (IFN-1) and pro-inflammatory cytokines, including tumor necrosis factor-alpha (TNF-α), interleukin-1 (IL-1), IL-6, and IL-18 [15-16]. The multifactorial etiology of organ dysfunction in sepsis results in a diverse array of clinical manifestations, often characterized by subtle and nonspecific signs and symptoms [17-18]. SA-AKI is an acute renal impairment induced by infection and may be accompanied by a syndrome of multi-organ dysfunction. The prognosis of SA-AKI is determined by the severity of AKI and the pre-existing organ reserve. Recovery from AKI depends on the regenerative capacity of endothelial and tubular cells, and a subset of patients may progress to chronic kidney disease [19-20]. Notably, patients requiring long-term hemodialysis have a 40-fold increased risk of developing sepsis [21]. To date, no specific biomarker for SA-AKI has been identified. Therefore, the identification of reliable biomarkers to aid in clinical decision-making remains a critical unmet need.

In this study, bioinformatics tools were employed to identify key genes associated with SA-AKI. We conducted a secondary analysis of the AKI sequencing dataset GSE225192 downloaded from the Gene Expression Omnibus (GEO) database and found that, compared with the non-AKI group, the expression levels of tumor necrosis factor receptor‑associated factor (TRAF)‑interacting protein with forkhead‑associated domain (TIFA) and interleukin-1 beta (IL-1β) were upregulated in the AKI group. We hypothesized that TIFA and IL-1β may be key genes in the pathogenesis of SA-AKI. Subsequently, we investigated the expression levels of TIFA and IL-1β in the peripheral plasma of patients with SA-AKI and analyzed the correlation between TIFA, IL-1β, and the occurrence of SA-AKI. The purpose of this study was to provide preliminary evidence for the potential of TIFA and IL-1β as diagnostic biomarkers for SA-AKI.

## Materials and methods

Data acquisition and differential gene screening

The dataset was obtained from the GEO database. In the dataset GSE225192, the gene expression profiles of renal tissues were compared between 14 rats with cecal ligation and puncture (CLP) and 14 sham-operated mice. Differential gene expression analysis was performed using the edgeR package, with the criteria of p<0.05 and |log2 fold change (FC)| ≥1. Subsequently, a volcano plot was generated to visualize the differentially expressed genes (DEGs). Furthermore, gene ontology (GO) functional enrichment analysis and Kyoto Encyclopedia of Genes and Genomes (KEGG) pathway analysis were conducted to elucidate the biological functions and pathways associated with these DEGs.

Study population

This was a prospective observational study. We recruited adult patients with sepsis according to the Sepsis 3.0 criteria and stratified them into groups based on the Kidney Disease: Improving Global Outcomes (KDIGO) criteria for AKI. The study data were derived from 105 patients with sepsis admitted to the ICU of the First Affiliated Hospital of Xinjiang Medical University between March 1, 2024, and December 1, 2024. A 28-day follow-up was conducted via telephone. All participants provided written informed consent. The study was approved by the Ethics Committee of Xinjiang Medical University (approval number: K202402-07).

Inclusion and exclusion criteria

Inclusion Criteria

The inclusion criteria were as follows: patients who met the diagnostic criteria for Sepsis-3, defined as the presence of a positive or suspected infection [4], and had a Sequential Organ Failure Assessment (SOFA) score of ≥2; patients aged ≥18 years and ≤85 years, regardless of gender; patients admitted to the ICU for >48 hours; and patients who provided written informed consent.

Exclusion Criteria

The inclusion criteria were as follows: patients younger than 18 years of age; pregnant or breastfeeding individuals; presence of pre-existing renal diseases (eg, nephrotic syndrome, lupus nephritis, interstitial nephritis, end-stage renal disease, etc); history of renal transplantation; presence of malignancies or hematological disorders; obstructive urinary tract disease; an estimated survival time of less than 48 hours; and a history of allergy to antibiotics or other medications.

Collection of clinical data and samples

This involved the collection of basic patient information (age, sex, contact telephone number), as well as clinical data such as the Acute Physiology and Chronic Health Evaluation II (APACHE II) score and SOFA score. Within 24 hours of admission, 5 ml of venous blood was collected from each patient. The blood samples were subsequently centrifuged, and the plasma was stored at -80 °C. Enzyme-linked immunosorbent assay (ELISA) assays were then performed on the stored plasma samples. The levels of TIFA and IL-1β in plasma were measured according to the instructions provided with the IL-1β ELISA kit (Wuhan Elabscience Biotechnology Co., Ltd., Catalog No: E-EL-H0149) and the TIFA ELISA kit (Jiangsu Enzyme Immune Industry Co., Ltd., Catalog No: MM-63547H1).

Statistical analysis

For the dataset (GSE225192), differential gene expression analysis was conducted using the edgeR package. A volcano plot was generated to visualize the DEGs. Clinical data were analyzed using STATA version 16.0. Continuous data are presented as mean ± standard deviation (SD). For comparisons between two groups, normally distributed data were analyzed using Student’s t-test, while non-normally distributed data were analyzed using the Wilcoxon test. Categorical data are presented as frequencies and proportions. The diagnostic value of TIFA and IL-1β across different groups was evaluated using receiver operating characteristic (ROC) curves. Correlation analysis was performed using Pearson’s correlation, with p<0.05 indicating statistical significance.

## Results

Transcriptomic analysis of sepsis module-trait association studies

**Figure 1:**
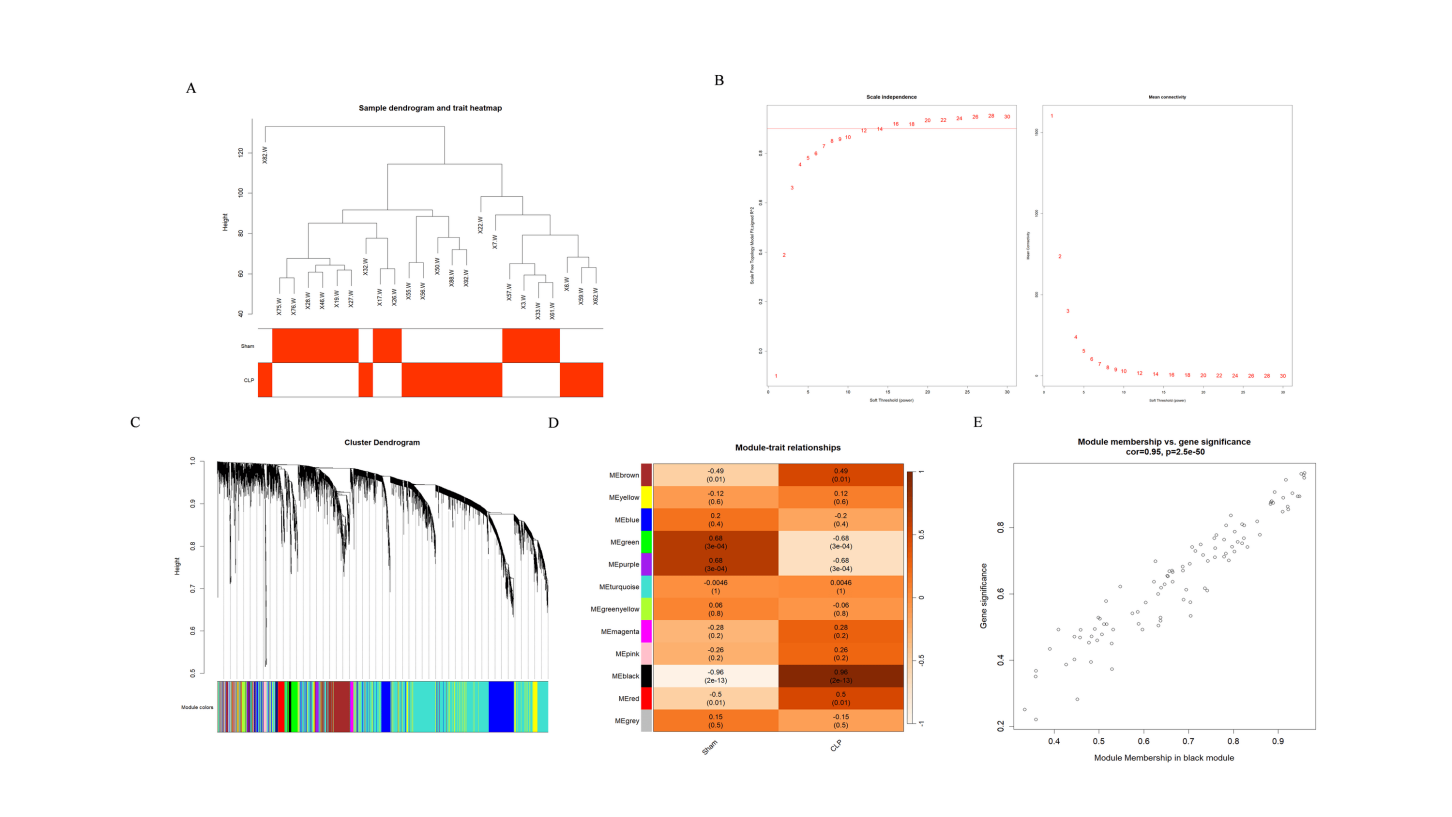
Transcriptome analysis of sepsis module-trait association study (A) The dendrogram displays the hierarchical clustering results of all samples. (B) The optimal soft threshold for WGCNA network construction: 8. On the left is scale independence, and on the right is mean connectivity. (C) The clustering dendrogram displays the clustering relationships among samples. (D) The module-trait relationship heatmap displays the correlations between different modules (such as black, brown, blue, green, etc.) in WGCNA and clinical phenotypes. (E) The scatter plot displays the correlation between module membership and gene significance, with module membership on the x-axis and gene significance on the y-axis WGCNA: weighted gene co-expression network analysis

In this study, we systematically explored the associations between sepsis gene modules and clinical phenotypes by integrating transcriptomics and weighted gene co-expression network analysis (WGCNA). As shown in Figure 1A, the sample dendrogram from hierarchical clustering revealed the heterogeneous grouping patterns of sepsis patients based on gene expression profiles, which corresponded significantly with clinical phenotypic characteristics, such as disease severity and organ dysfunction. Based on the soft-thresholding analysis(Figure 1B), the optimal parameter β=8 (R²=0.96) was determined, successfully constructing a co-expression network that met the scale-free topology criteria. Gene clustering analysis (Figure 1C) identified 12 highly co-expressed gene modules, among which the black module (n=98 genes) exhibited significant phenotypic associations (Figure 1D). Module-trait correlation analysis showed that the black module was strongly positively correlated with the APACHE II score (r=0.96, p=2.e-13), suggesting its potential involvement in the pathological damage and immune regulation processes of sepsis. The scatter plot (Figure 1E) demonstrated the positive correlation between module membership and gene significance, indicating that the expression levels of module members were associated with the extent of phenotypic traits. This study is the first to elucidate the modular structure characteristics of sepsis-specific gene co-expression networks, providing an important theoretical basis for deciphering its molecular subtyping, discovering therapeutic targets, and identifying prognostic biomarkers.

Expression differences of sepsis-related genes across renal tissues

**Figure 2:**
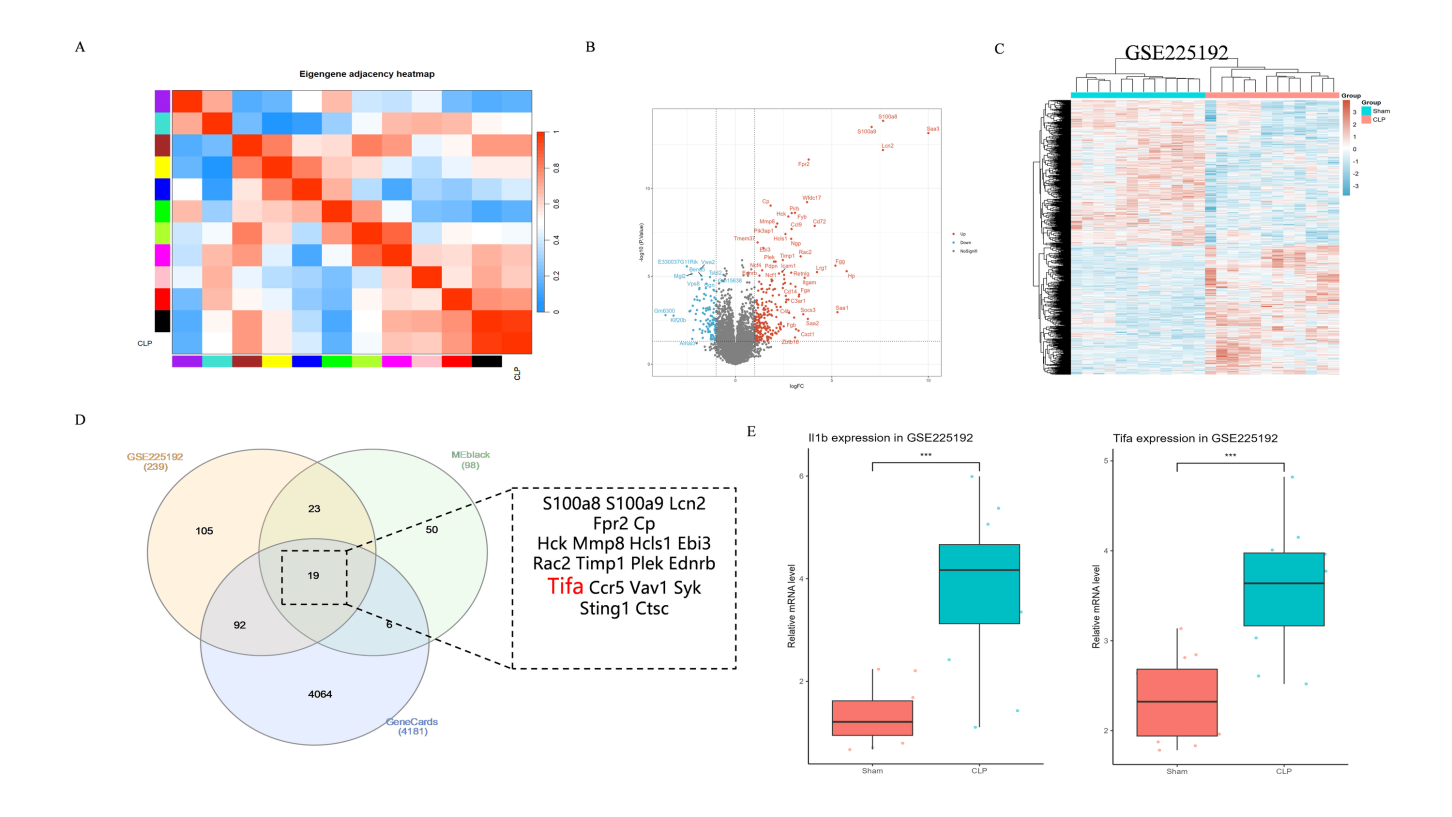
Analysis of sepsis-related gene modules and their expression differences in renal tissue (A) The gene adjacency heatmap displays gene expression in samples, with different colors representing high or low levels of module gene expression. (B-C) Volcano and heatmap plots display differential gene expression in renal tissue between sham and CLP in GSE225192, with criteria of p<0.05 and |Log2FC| >1.0. (D) Venn diagrams and gene lists display overlapping genes across different datasets (GSE225192, Meblack, and the GeneCards database). (E) The expression differences of TIFA and IL-1β mRNA in renal tissues between sham and CLP are shown via the box plot CLP: cecal ligation and puncture; IL-1β: interleukin-1 beta; tumor necrosis factor receptor‑associated factor‑interacting protein with forkhead‑associated domain

In the analysis of sepsis-related gene modules, differential expression of genes across various groups revealed co-expression patterns among genes in different modules, with the highest gene expression observed in the CLP group (Figure 2A). Differential analysis identified multiple genes that exhibited significant changes between the CLP and sham groups, including S100A8, S100A9, TIFA, and Ccr5. A total of 239 genes were upregulated, and 104 genes were downregulated (Figures 2B, 2C). These genes may play important roles in the development and progression of sepsis. Box plots were used to illustrate the significant differences in TIFA and Il-1β gene expression between the CLP and sham groups in renal tissues.

Baseline characteristics of the study population

The study commenced in March 2024 and concluded in December of the same year. A total of 105 patients were initially selected for inclusion. Based on the exclusion criteria, 10 patients were excluded (five with a prior diagnosis of AKI, two with chronic kidney disease, and three who refused all treatments). Ultimately, 95 patients (90.1%) were enrolled in the study. Among them, 47 patients were diagnosed with AKI, 22 died due to AKI, and three died from causes other than AKI. The demographic and baseline characteristics of the patients, stratified by AKI status, are presented in Table 1. There were 47 male (49%) and 48 female patients (51%). No significant differences were observed between groups in terms of gender (p=0.759). Patients in the AKI group had a mean age of 62.13 years, which was significantly higher than that in the non-AKI group (54.65 years; p=0.013). There were no significant differences between the two groups in terms of gender ratio, neutrophil percentage, albumin, globulin, or albumin-to-globulin ratio. SOFA and APACHE II scores were significantly higher in the AKI group compared to the non-AKI group ( p<0.001). These data suggest that older age, higher white blood cell count, elevated serum creatinine levels, higher SOFA and APACHE II scores, and increased levels of IL-1β and TIFA may be associated with an increased risk of AKI.

**Table 1:** Patients demographics and clinical characteristics (n=95) based on the presence of AKI ^*^The variable data follow a normal distribution per the t-test. ^**^The variable data do not follow a normal distribution per the rank-sum test AKI: acute kidney injury; APACHE II: Acute Physiology and Chronic Health Evaluation II; IL-1β: interleukin-1 beta; SD: standard deviation; SOFA: Sequential Organ Failure Assessment; TIFA: tumor necrosis factor receptor‑associated factor‑interacting protein with forkhead‑associated domain

Characteristics	AKI (n=47）	Non-AKI (n=48）	t/Z	P-value
Age^**^, years, mean ± SD	62.13 ± 12.87	54.65 ± 15.75	-2.530	0.013
Male sex^**^, n (%）	24 (51.06)	23 (47.92)	0.094	0.759
WBC count^*^ × 10⁹/L, median (Q1, Q3)	13.56 (11.39, 18.39)	12.08 (10.81, 14.12)	-2.002	0.0452
Neutrophil percentage^*^, mean ± SD	85.02 ± 7.62	84.01 ± 8.10	-0.620	0.5363
Serum creatinine day 0^*^, mg/dL, median (Q1, Q3)	95.93 (68, 14)	74 (54.05, 95.97)	-1.932	0.0534
Serum creatinine day 2^*^, mg/dL, median (Q1, Q3)	142 (104, 203)	68 (55.2, 84.50)	-6.767	<0.001
Albumin^*^, g/L, median (Q1, Q3)	32 (29.8, 34.22)	32.2 (30.14, 33.44)	0.462	0.644
Globulin^*^, g/L, median (Q1, Q3)	33 (30.16, 35.30)	32.62 (30.31, 36.47)	0.220	0.826
Albumin-to-globulin ratio^*^, %, median (Q1, Q3)	0.95 (0.88, 1.03)	0.995 (0.89, 1.07)	0.946	0.344
Serum lactic acid^*^, mmol/L, median (Q1, Q3)	2.3 (1.30, 3.30)	1.7 (1.2, 2.25)	-1.852	0.064
Length of stay^*^, days, median (Q1, Q3)	13 (10, 25)	12 (9, 18)	-0.917	0.359
SOFA score^*^, median (Q1, Q3)	11 (9, 13)	5 (4, 6)	-6.404	<0.001
APACHE II score^*^, median (Q1, Q3)	23 (19, 25）	14 (11.50, 17.50)	-5.624	<0.001
IL-1β^*^, pg/ml, median (Q1, Q3)	30.20 (10.13, 50.24)	5.544 (3.35, 10.36)	-5.665	<0.001
TIFA^*^, pg/ml, median (Q1, Q3)	430.78 (174.21, 526.65)	102.88 (82.81, 120.30)	-6.290	<0.001

Plasma expression levels of TIFA and IL-1β

**Figure 3:**
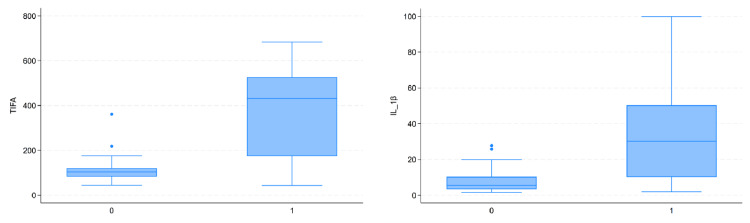
Plasma expression levels of TIFA and IL-1β (A) The expression differences of plasma TIFA in AKI and non-AKI patients are shown. (B) The expression differences of plasma IL-1β in AKI and non-AKI patients are shown. "0" represents non-AKI patients, and "1" represents AKI patients AKI: acute kidney injury; IL-1β: interleukin-1 beta; TIFA: tumor necrosis factor receptor‑associated factor‑interacting protein with forkhead‑associated domain

The IL-1β and TIFA genes, which were identified as upregulated in the AKI group through bioinformatics analysis, were selected for further validation. The plasma levels of IL-1β and TIFA were measured using ELISA. The results demonstrated that, compared with the non-AKI group, the plasma levels of IL-1β and TIFA were significantly elevated in the AKI group (p<0.001 for both) (Figure 3). These findings corroborated the bioinformatics analysis, highlighting the potential role of these inflammatory mediators in AKI pathogenesis.

Correlation between TIFA, IL-1β, and APACHE II scores

**Figure 4:**
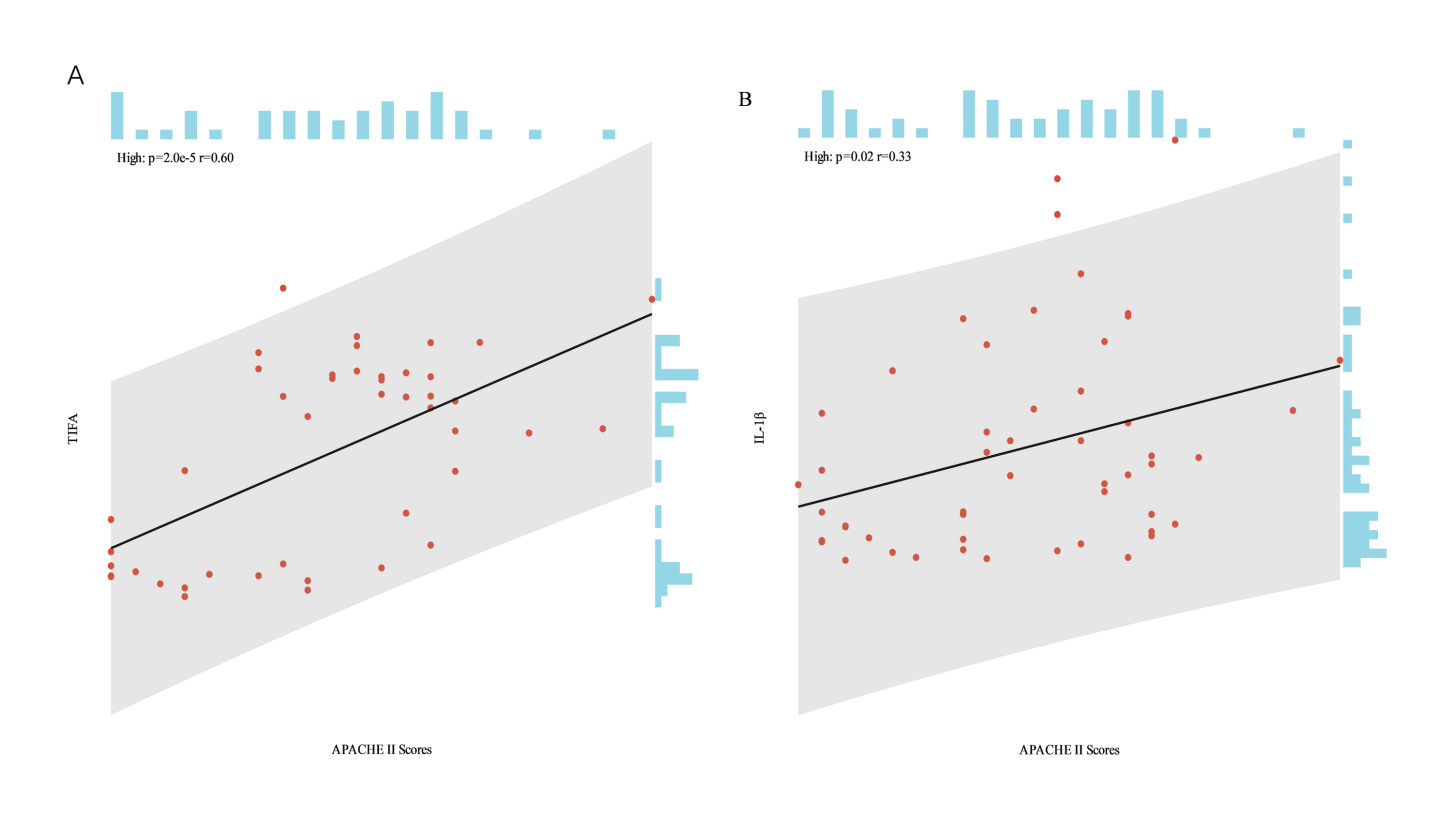
Correlation between TIFA, IL-1β, and APACHE II scores (A) Correlation analysis between TIFA and APACHE II scores. (B) Correlation analysis between IL-1β and APACHE II scores APACHE II: Acute Physiology and Chronic Health Evaluation II; IL-1β: interleukin-1 beta; TIFA: tumor necrosis factor receptor‑associated factor‑interacting protein with forkhead‑associated domain

We assessed the relationship between plasma levels of TIFA and IL-1β and the APACHE II score in patients with SA-AKI using Pearson correlation analysis (Figure 4). Our data demonstrated that plasma levels of TIFA and IL-1β positively correlated with the APACHE II score (r=0.60, p<0.01 for TIFA; r=0.33, p=0.02 for IL-1β).

The clinical value of TIFA and IL-1β in diagnosing SA-AKI

**Figure 5:**
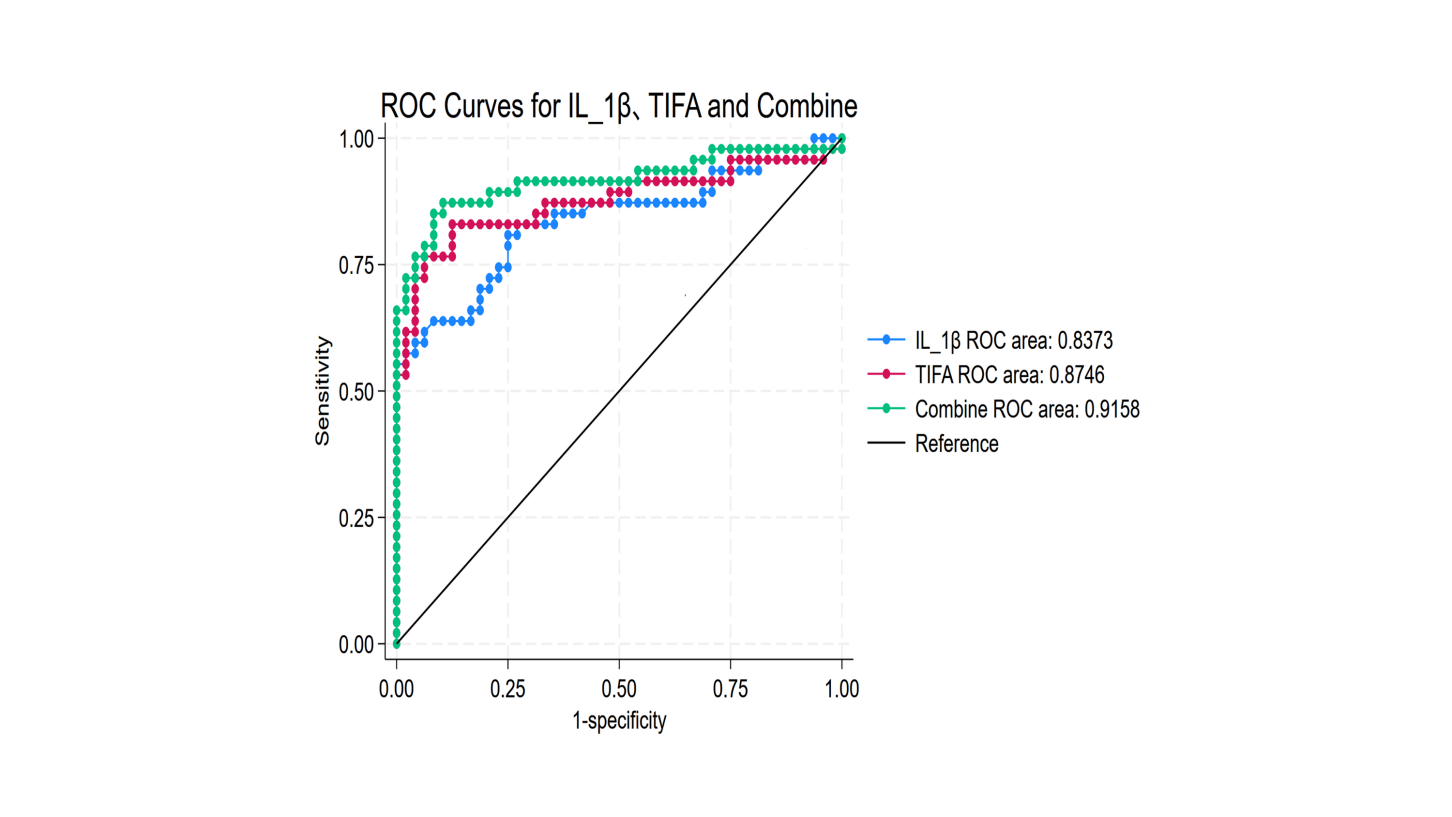
ROC curves for IL-1β and TIFA predict SA‐AKI risk IL-1β: interleukin-1 beta; ROC: receiver operating characteristic; SA‐AKI: sepsis-associated acute kidney injury; TIFA: tumor necrosis factor receptor‑associated factor‑interacting protein with forkhead‑associated domain

To further investigate the value of IL-1β and TIFA in predicting the risk of SA-AKI, we conducted ROC curve analyses. Figure 5 presents the ROC curves, while Table 2 provides detailed diagnostic criteria for each biomarker, including the area under the ROC curve (AUC), Youden's index, sensitivity, specificity, confidence intervals (CI), and cutoff values. When the cutoff value for plasma IL-1β was set at 8.47 pg/mL, its AUC for predicting AKI in sepsis patients was 0.837 (95% CI: 0.754-0.921). For TIFA, with an optimal cutoff value of 137.256 pg/mL, the AUC for predicting AKI in sepsis patients was 0.874 (95% CI: 0.796-0.953). The combined prediction of AKI using both plasma IL-1β and TIFA achieved an AUC of 0.916 (95% CI: 0.852-0.979), which was significantly higher than that of either biomarker alone. The combined prediction demonstrated a sensitivity of 85.11% and a specificity of 91.67%. These results highlight the potential of plasma IL-1β and TIFA as diagnostic biomarkers for SA-AKI, suggesting that their combined use may improve the accuracy of predicting AKI in sepsis patients.

**Table 2:** Diagnostic performance of TIFA and IL‐1β for SA‐AKI AUC: area under the receiver operating characteristic curve; CI: confidence interval; IL-1β: interleukin-1 beta; SA‐AKI: sepsis-associated acute kidney injury; TIFA: tumor necrosis factor receptor‑associated factor‑interacting protein with forkhead‑associated domain

Biomarkes	AUC	Cutoff	Sensitivity (%)	Specificity (%)	Youden's index (%)	95% CI
IL-1β	0.8373	8.465	82.98%	72.92%	62.90%	0.75382–0.92083
TIFA	0.8746	137.256	82.98%	87.50%	70.48%	0.79646–0.95266
Combined	0.9158	-	85.11%	91.67%	76.78%	0.85224–0.97932

## Discussion

SA-AKI is a severe but potentially reversible complication associated with high morbidity and mortality. Compared with sepsis patients who do not develop AKI, those with SA-AKI have a worse long-term prognosis and incur higher costs [22]. The pathogenesis of SA-AKI remains incompletely understood. Moreover, we are currently unable to directly monitor and manage renal blood flow (RBF), renal parenchymal inflammation, and changes in immune function, as well as cortical and medullary perfusion and oxygenation, microcirculatory flow and oxygenation, and tubular epithelial cell health-all of which significantly influence renal function [23-25].

Predicting and identifying patients at risk of developing SA-AKI is crucial for formulating appropriate therapeutic plans. TIFA, as a novel biomarker, may aid in the differential diagnosis of SA-AKI and the early detection of disease progression. Its integration with corresponding therapeutic strategies could be valuable in ICU clinical practice. The caspase-1 inhibitor AC-YVAD-CMK has demonstrated renal protective effects in a CLP rat model [26]. In addition to conventional indicators such as serum creatinine, urine output, SOFA score, and APACHE II score for predicting the occurrence of SA-AKI, we have found that maintaining fluid balance helps identify patients at high risk of AKI [27]. However, the sensitivity and specificity of these indicators are inconsistent. Inflammatory markers play a vital role in the diagnosis and treatment of sepsis [28-29].

In our search for novel biomarkers to predict disease, we performed analyses of DEGs, KEGG pathway analysis, and GO analysis on the dataset GSE225192 from the GEO database. Our secondary analysis of the GSE225192 dataset revealed that, compared with non-AKI mice, renal tissues from AKI mice exhibited upregulated mRNA expression of TIFA and IL-1β. These findings highlight an imbalance in inflammatory responses in the kidneys of mice with AKI.

TIFA is an adaptor protein involved in both adaptive and innate immunity. The C-terminus of TIFA contains a forkhead-associated (FHA) domain, which recognizes phosphorylated serine or threonine residues. The N-terminus of TIFA has a threonine-binding site that, upon phosphorylation by alpha kinase 1 (ALPK1) at threonine residue 9, enables TIFA to form head-to-tail dimers in an antiparallel orientation. These structures are termed TIFA bodies [30]. TIFA may participate in the regulation of innate immune responses via the TIFA-TRAF signaling pathway. LPS from Gram-negative bacteria binds to ALPK1, a recently identified pattern recognition receptor (PRR), which subsequently activates TIFA through phosphorylation and leads to the nuclear factor kappa-light-chain-enhancer of activated B cells (NF-κB) activation [31]. In one study, compared with control cells, LPS-stimulated cells overexpressing TIFA exhibited significantly enhanced transcriptional activation of DNA damage-induced genes and secretion of the canonical NF-κB pathway, including IL-1β [32-33]. TIFA activation of the NF-κB signaling pathway has been implicated in periodontitis in diabetic mice, with increased expression of IL-1β and IL-18 [34].

We selected TIFA and IL-1β for clinical validation and evaluated their potential as biomarkers through predictive modeling. Our analysis of the dataset (GSE225192) revealed that, compared with the sham group, mRNA levels of TIFA and IL-1β were significantly elevated in sepsis. In clinical experiments, we measured plasma levels of TIFA and IL-1β in patients with sepsis. Results showed that, compared with non-AKI patients, levels of TIFA and IL-1β were significantly higher in patients with AKI. We speculate that this may be related to the involvement of TIFA and IL-1β in the pathogenesis of SA-AKI. As sepsis progresses, further tubular injury occurs, leading to progressive renal dysfunction. To further confirm the importance of TIFA and IL-1β in the pathogenesis of SA-AKI, we performed ROC curve analysis, which demonstrated that TIFA and IL-1β have high value in differentiating SA-AKI, with the highest AUC achieved when used in combination. Zhuang et al. found that delayed initiation of continuous renal replacement therapy (CRRT) in patients with SA-AKI is associated with improved survival, although it is not a definitive factor, highlighting the need for more personalized treatment strategies [35]. Interestingly, we also found that male sex may be an independent risk factor for AKI in sepsis patients, potentially related to hormonal secretion [36].

**Figure 6:**
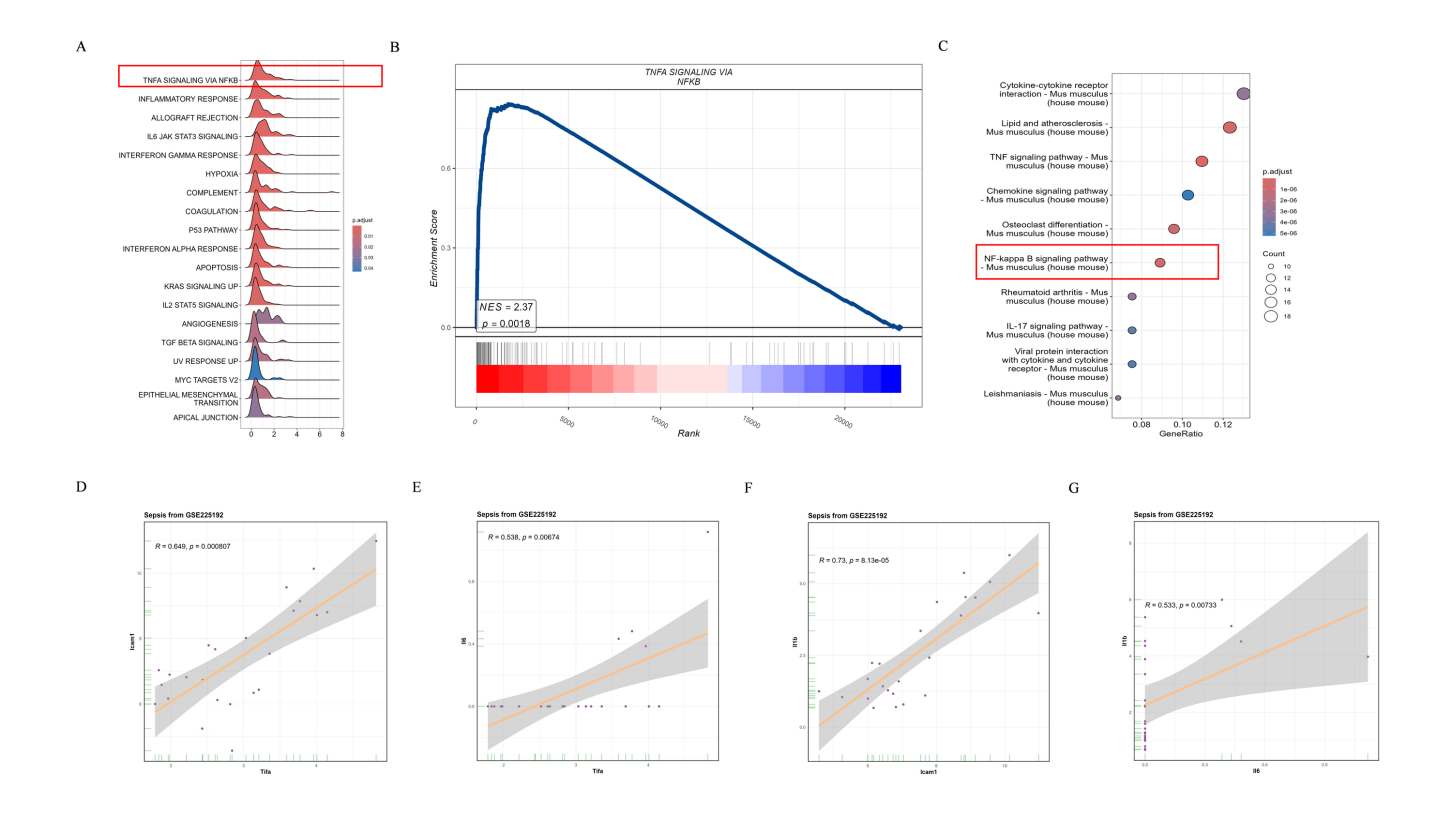
Enrichment analysis of the NF-κB signaling pathway in sepsis (A) The ridge plot displays the ranking of significant pathways in gene set enrichment analysis, where the NF-κB signaling pathway is prominently enriched. (B) The gene set enrichment score displays the enrichment score curve of gene sets in the NF-κB signaling pathway. (C) KEGG analysis displays the significance analysis of various pathways; the NF-κB signaling pathway is significantly enriched. (D-G) Correlation analysis displays the correlation between the gene expression of TIFA, IL-1β, and the NF-κB pathway IL-1β: interleukin-1 beta; KEGG: Kyoto Encyclopedia of Genes and Genomes; NF-κB: nuclear factor kappa-light-chain-enhancer of activated B cells; TIFA: tumor necrosis factor receptor‑associated factor‑interacting protein with forkhead‑associated domain

Additionally, through secondary analysis of the dataset, we identified that TIFA activation may be associated with the NF-κB signaling pathway. In the enrichment analysis of the NF-κB signaling pathway in sepsis, we observed significant enrichment in gene set enrichment analysis (Figure 6A), suggesting that this pathway may play a critical role in the pathogenesis of sepsis. The gene set enrichment score curve (Figure 6B) further confirmed the significant enrichment of genes within the NF-κB signaling pathway, with a median absolute deviation (MAD) of 0.35, indicating substantial changes in gene expression within this pathway. The KEGG analysis (Figure 6C) also highlighted the significance of various pathways, with the NF-κB signaling pathway being prominently enriched, thereby further supporting our findings. Additionally, correlation analysis (Figure 6D-6G) revealed positive correlations between the expression of TNFα, IL-1β, and genes in the NF-κB signaling pathway, with correlation coefficients of 0.53 for TNFα and 0.56 for IL-1β. These results suggest that these inflammatory cytokines may contribute to the inflammatory response in sepsis through activation of the NF-κB signaling pathway. Previous studies have shown increased expression of NF-κB in sepsis-induced lung injury [37]. Several studies have reported widespread activation of NF-κB in patients with sepsis, indicating that inhibition of the NF-κB signaling pathway may be a potential therapeutic target [38-41].

## Conclusions

TIFA may serve as an early predictive marker for SA-AKI. Several studies have shown a significant association between TIFA and the development of SA-AKI, with its expression levels potentially identifying high-risk sepsis patients before the onset of overt clinical symptoms, thereby providing a window for early intervention and treatment. Compared with some traditional diagnostic methods or biomarkers for SA-AKI, TIFA may exhibit higher sensitivity and specificity. Its performance in predicting the occurrence of SA-AKI appears superior to that of commonly used clinical scoring systems or other biomarkers, suggesting that it may more accurately identify true-positive and true-negative cases. However, whether the activation of TIFA in SA-AKI leads to IL-1β production through the involvement of other inflammatory mediators remains unknown. We hope to elucidate the pathogenesis of SA-AKI through multicenter studies with larger sample sizes. In this study, we measured only the plasma levels of TIFA and IL-1β and did not conduct any additional investigations. Hence, our findings should be interpreted with caution. Future studies should consider establishing animal models of SA-AKI to conduct relevant experiments. Additionally, it is important to emphasize that our study assessed the levels of TIFA and IL-1β in plasma, which may not accurately correlate with mRNA levels. Thus, our results do not directly indicate increased mRNA expression of these biomarkers.
